# DeepRice6mA: A convolutional neural network approach for 6mA site prediction in the rice Genome

**DOI:** 10.1371/journal.pone.0325216

**Published:** 2025-06-18

**Authors:** Hussam Alsharif

**Affiliations:** Jamoum University College, Computer Science Department, Umm Al-Qura University, Makkah, Saudi Arabia; China Three Gorges University, CHINA

## Abstract

As one of the most critical post-replication modifications, N6-methylation (6mA) at adenine residue plays an important role in a variety of biological functions. Existing computational methods for identifying 6mA sites across large genomic regions tend to fall short in either accuracy or computational efficiency. To address this, we introduce DeepRice6mA, a sophisticated comprehensive predictive tool for identifying rice 6mA sites, using a deep learning approach that incorporates ensemble strategies from one-hot encoding and 3-kmer feature embedding. The proposed model, labeled DeepRice6mA, reaches state-of-the-art results compared to current approaches, with 10-fold cross-validation scores of 98% for accuracy, 98% for sensitivity, 98% for specificity, a Matthew’s correlation coefficient (MCC) of 0.96, and an area under the receiver operating characteristic curve (AUC) of 0.99. We anticipate that DeepRice6mA will significantly enhance our understanding of DNA methylation and its implications for biological processes and disease states.

## Introduction

**Fig 1:**
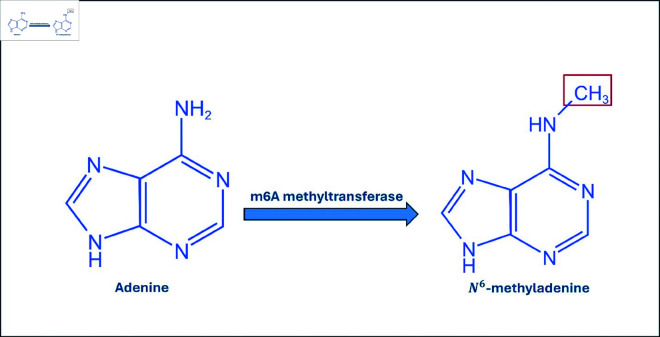
The modifications of N6-methyladenine (6mA) in DNA. The process of converting adenine to 6mA is carried out by methyltransferase enzymes.

There are many kinds of epigenetic modifications in DNA, including N4-methylcytosine, N6-methyladenine, and 5-methylcytosine, which occur in diverse organisms [1]. A large number of modifications, such as DNA 6-methylation performed by methyltransferases targeting DNA molecules, exist [2]. Homologous recombination occurs after DNA replication. This change has been found across all three categories of organisms, which include eukaryotes, bacteria, and archaea [3]. Previous studies by our team demonstrated that N6 methylation is the addition of a methyl group to the sixth position of the purine ring in adenine nucleotides. Importantly, this modification serves as an important mechanism to differentiate between parental and newly synthesized DNAs; hence, it plays a central role in gene transcription, replication, and repair processes [4, 5]. Fig 1 illustrates the modifications of N6-methyladenine (6mA) in DNA, highlighting the process by which adenine is converted to 6mA by methyltransferase enzymes.

Several laboratory methodologies, including sequencing techniques like SMRT-seq and MeDIP-seq [6], as well as methylated DNA immunoprecipitation [7], capillary electrophoresis, and laser-induced fluorescence [8], have been suggested for detecting N6-methyladenine (6mA) sites within the genome. The N6-methyladenine profile of rice has recently been characterized by mass spectrometry analysis and N6-methyladenine immunoprecipitation, along with sequencing of the immunoprecipitated samples [9]. However, recognizing 6mA sites across the genome using these experimental approaches is labor-intensive and costly. A computational analysis using a pLogo plot [10], based on a standard dataset for identifying 6-methyladenine (6mA) sites in rice, demonstrates that the distribution of nucleotides near 6mA sites differs compared to that near non-6mA sites [11]. Consequently, reliable computational approaches can be very effective in pinpointing 6mA sites.

The advancement of practical methods will assist in the study of 6mA changes, particularly through techniques such as mass spectrometry for correct identification, immunoprecipitation for isolating modified DNA, and high-throughput sequencing for mapping. These methods collectively provide valuable insights into the roles and impacts of 6mA in genetic regulation and epigenetics. In the meantime, computational predictions of DNA 6mA sites within a genome have become a feasible alternative due to the limitations associated with laborious and expensive experimental trials. In recent times, the adoption of investigative methods utilizing machine learning and deep learning approaches has addressed many challenges in identifying 6mA modifications. The 6mA modification remains a subject of considerable interest in research, with many scientists employing machine learning and deep learning methods to recognize 6mA sites in the rice genome [12, 13].

Two types of benchmark datasets were used for all existing applications. The first dataset was used for seven methods, which contained 880 samples for each class [14–20]. These methods were as follows:

The first method, i6mA-Pred [14], was used to identify 6mA sites in the rice genome. It was developed using pseudo amino acid composition (PseAAC) as a feature representation and applied with a support vector machine (SVM).

The second method, 6mA-RicePred [15], is a tool that incorporates three key features: binary encoding, k-mer, and Markov. These features are utilized in combination with a support vector machine (SVM) to identify 6mA sites.

The third method, iDNA6mA [16], is based on one-hot encoding and utilizes a one-dimensional (1D) convolutional neural network (CNN) to predict 6mA site.

The fourth method, SNNRice6mA [17], is built using one-hot encoding and includes several normalization layers between the convolutional layers, along with an adaptive learning rate.

The fifth method, i6mA-DNCP [18], constructed two features: dinucleotide composition frequency and dinucleotide-based DNA types. This method applied feature selection over the constructed features with the Tree Bagging algorithm as a classifier.

The sixth method, SDM6A [19], used nine encoding features, applied feature selection to extract the best features, and employed two integrated classifiers, the support vector machine (SVM) and an extremely randomized tree (ERT).

The seventh method, i6 mA-CNN [20], is a recent method for detecting 6mA sites in the rice genome. It employed several features, such as pseudo amino acid composition (PseAAC), multiple one-hot representations, and dinucleotide physicochemical properties, to build a customized feature vector. The classifier used for these features is a one-dimensional (1D) convolutional neural network (CNN) to predict 6mA sites.

A second benchmark dataset of a rice genome containing 154,000 samples per class was used [21–25]. The methods applied using this dataset were as follows:

The first method, iDNA6 mA-Rice [21], constructed the one-hot vector feature space using the Random Forest classifier to predict 6mA sites.

The second method, MM-6mAPred [22], integrates neighbor dependency information with a Markov model to detect 6mA sites.

The third method, 6mA-Finder [23], utilized seven sequence features and three physicochemical features, applying the recursive feature elimination approach to obtain optimal features. It then employed these features in the Markov Model algorithm to detect 6mA sites.

The fourth method, DNA6mA-MINT [24], integrates one-hot encoding with two classifiers: convolutional neural network (CNN) and long short-term memory (LSTM) networks.

The fifth and most recent method, SpineNet-6mA [25], implements one-hot encoding and uses hybrid classifiers, including CNN and SpinalNet, to predict 6mA sites.

While the outcomes of these methods show promise, there is a risk of bias due to the manual selection of attributes, as well as the possibility of missing unknown attributes that could advance the identification of 6mA sites in the rice genome. Furthermore, the predictive performance of these methods has not yet reached a satisfactory level for application in large-scale genomic studies.

**Fig 2:**
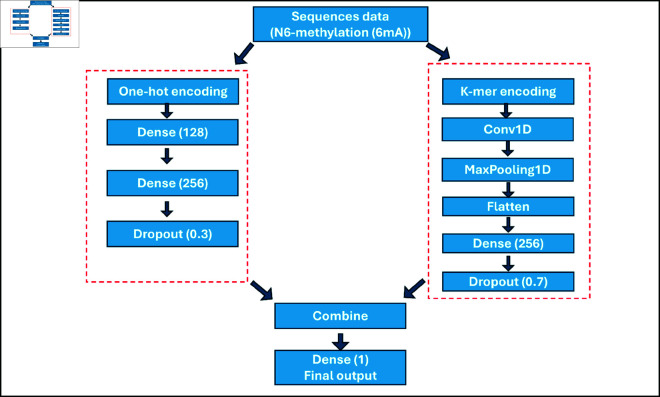
Architecture of DeepRice6mA.

In this work, we developed a computational method named “DeepRice6m” to identify 6mA sites within the rice genome. This method integrates strategies from one-hot encoding and 3-kmer feature embedding. Fig 2 illustrates the architecture of DeepRice6mA. Our model effectively corresponds to these features, allowing it to successfully distinguish between 6mA and non-6mA sites. We believe that the identification of frequently occurring motifs may be significant for conducting further research in this field. Experimental evaluations of two benchmark datasets show the effectiveness and robustness of our proposed tool.

## Datasets

The first dataset applied in this study is referred to as Dataset 1. This dataset has been widely used in various studies [14–20]. It contains sequences obtained from the National Center for Biotechnology Information (NCBI)Gene Expression Omnibus (GEO) with accession numbers. In this dataset, sequences that contained a centrally discovered 6mA modification and had an alteration score of more than 30 were characterized as positive samples. Here, a mark of 30 is considered the threshold for recognizing a nucleotide as altered. Every sequence in this dataset had a length of 41 base pairs. To remove redundancy from the positive sequences, the Cluster Database at High Identity with Tolerance (CD-HIT) [26] was used, with the threshold set to 60%. Consequently, we identified a total of 880 positive instances. Consistent 880 negative instances were collected using specific criteria. The first criterion was that the sequences be 41 base pairs long, alongside an adenine nucleotide at the central. The second criterion was the experimental verification that the central adenine was unmethylated. The third criterion involved selecting negative test samples to maintain a reliable proportion of the GAGG motif, similar to that found in the positive samples. This approach ensured that the fraction of the GAGG motif in the negative class was comparable to that in the positive class. The second dataset, referred to as Dataset 2, is also broadly recognized for its application in various approaches [20–25]. It was obtained from the Gene Expression Omnibus (GEO) database run by the NCBI and contains about 265,290 sequences, with each sequence measuring 41 base pairs in size, with 6mA sites positioned at the center . To improve this dataset, CD-HIT software [26] was applied, with a threshold of 80%, which matches with outcomes from other studies [20–25]. This method was designed to eliminate homologous sequences. Therefore, we created a positive sample containing 154,000 sequences that contained 6mA sites.

Negative samples were obtained from the NCBI [27] based on three standards: (1) the sequences must be 41 base pairs long, with an adenine nucleotide in the middle, (2) it has been experimentally verified that the central adenine is unmethylated, and (3) 6mA modifications typically happens proximity to the GAGG motif [9]. Moreover, negative samples were selected to contain a similar percentage of this motif among the negative class samples similar to that found in the positive samples.

As a result of the selection process, we collected many negative samples. To achieve a balanced dataset, we randomly selected 154,000 negative samples to match the number of positive samples.

## Sequence construction

An example sequence from our dataset is illustrated as follows:


R=R1,R2,……RN

Here, N represents the length of the DNA sequence, which is 41 in this instance. By employing a suitable statistical approach to the representations of these sequences, the deep learning models could identify the features that distinguished between 6mA sites and non-6mA sites based on both local and global sequence patterns [28]. In our analysis, we employed several statistical techniques for the representations of the DNA sequences, enhancing the capability of our deep learning models to identify the features that distinguish between 6mA sites and non-6mA sites.

Specifically, we utilized Principal Component Analysis (PCA), which was applied to reduce dimensionality, allowing us to extract the most significant features while preserving the variance in the data set. Additionally, we used the Chi-square test to evaluate the independence of features and their relevance to the target variable (6mA modification), helping to filter out less relevant features. We also used Recursive Feature Elimination (RFE) to recursively eliminate the least significant features and optimize the feature set for improved model performance. Furthermore, we applied Genetic Algorithms as an optimization technique to explore different combinations of characteristics and determine the most predictive subsets.

We analyzed the average feature importance derived from the hidden layers of our Convolutional Neural Network (CNN) models, and our findings indicated that using the entire feature set significantly improved the model’s ability to distinguish between the two classes, thereby demonstrating the power of CNNs in capturing intricate patterns from the full range of features.

By incorporating these statistical methods, we aimed to optimize the model’s predictive capabilities while ensuring robust classification of 6mA sites.

In our model, we utilized the following representations for the sequences:

### One-hot encoding

One-hot encoding transfers string elements into a binary format. We utilized one-hot similar to the methodology presented in this study [29]. The four common nucleotides were converted into numerical values ranging from 0 to 3. Each nucleotide was denoted by a binary sequence comprising a series of zeros with a single “1,” indicating the identity of the nucleotide. In our study, we assigned binary representations in alphabetical sequence. For instance, adenine (A) was encoded as 1000, cytosine (C) as 0100, guanine (G) as 0010, and thymine (T) as 0001. Consequently, in our method, a segment length L corresponds to a feature matrix of size L × 4. Therefore, the total number of features was 164.

Although one-hot encoding does not inherently differentiate between unmodified adenine and N6-methyladenine (6mA), we employed several strategies to establish this distinction during model training. Our model leverages contextual information surrounding the adenine nucleotide. Training in sequences that include known 6mA modifications, the model learns patterns associated with these modifications based on their positions relative to surrounding nucleotides, enabling it to differentiate between modified and unmodified adenine.

In addition to one-hot encoding, we utilized k-mer encoding with a size of (k = 3). This technique captures sequences of three nucleotides at a time, allowing the model to analyze local patterns indicative of 6mA modifications. By examining overlapping triplets, the model can effectively identify sequence motifs that correlate with these modifications. The training incorporated labeled data that feature known 6mA modifications, allowing the model to learn critical features related to these modifications. The one-hot representation is complemented by the enriched information obtained from the broader context provided by the k-mer encoding.

Furthermore, our deep learning architecture allows the model to capture intricate relationships and interactions between features, which are essential for reliably predicting 6mA sites. This layered learning capability is a hallmark of deep learning and enhances model performance. To evaluate effectiveness, we employed 10-fold cross-validation, ensuring that our approach is robust and generalizable.

One disadvantage of one-hot encoding is that it creates a uniform representation, which means that nucleotides with similar properties may not be categorized together in the vector space. One-hot encoding produces a uniform vector representation of nucleotides, meaning that each nucleotide is treated as distinct and separate from the others. As a result, nucleotides that share similar chemical or biological properties, such as polarity or base pairing behavior, are effectively classified independently from each other. For example, adenine and guanine are purines, sharing structural similarities, but are represented as entirely distinct vectors (1000 for A and 0010 for G).

The binary nature of one-hot encoding does not capture the relationships or similarities between the nucleotides and does not reflect any inherent properties that the nucleotides might share. A nucleotide with particular properties (such as being a purine or pyrimidine) will not have any numeric correlation to another nucleotide with the same property, which can lead to suboptimal performance when building models that may benefit from such relationships.

The property referenced here refers to various biochemical characteristics of nucleotides, such as chemical structure, hydrophobicity or hydrophilicity, and base pairing behavior. Nucleotides can be classified into two structural categories: purines which include adenine and guanine and pyrimidines which consist of cytosine and thymine. This characteristic affects their base pairing and biochemical interactions. Different nucleotides exhibit varying levels of hydrophobicity or hydrophilicity, which can influence the way they interact with other molecules in the cellular environment. The ability of nucleotides to pair with each other during DNA and RNA synthesis is fundamental to their function, and this pairing is determined by their structural attributes.

### Embedding layer

Embedding is a procedure used to convert distinct variables into continuous vector representations. It generates a dense vector with a determined number of dimensions that can be chosen arbitrarily. One of the most well-recognized applications of embedding is characterizing the vocabulary of documents via word embedding. According to Collobert *et al*. [30], word embeddings can discover numerous hidden relations amongst phrases. Embedding layer techniques have been used in many studies of DNA and protein problems [31–34]. We employed embedding coding to denote nucleotide sequences more efficiently, similar to the method described in previous work [34], while tackling the constraints of one-hot encoding. Initially, the four basic nucleotides (A, C, G, and T) were transformed into integer values that scaled from 0 to 3. This numerical representation was then fed into the embedding layer, which operated as the first layer in the deep learning architecture. The embedding layer was initialized with random weights, allowing it to discover the best vector-based interpretations during training over several epochs. This approach enables the model to dynamically learn the best representations of the input sequences, which proves particularly beneficial because it captures nuanced relationships between different nucleotides, offering a more flexible representation compared to one-hot encoding.

Random weights are initialized randomly at the start of the training process and adjusted through backpropagation as the model learns. This adjustment allows the model to optimize its representation based on training data, effectively uncovering latent features that distinguish nucleotide modifications.

Weighted properties refer to weights that have been trained and optimized based on the input data, reflecting the importance of each feature. In contrast, unweighted representations utilize fixed, predefined values without optimization. For example, if all weights are set to a constant value, it restricts the model’s ability to learn or adapt to the data.

Performance validation using metrics such as accuracy and sensitivity allows us to assess how effectively the model distinguishes between classes using weighted and unweighted approaches. This comparative analysis elucidates the advantages of employing random, trainable weights in our embedding layer and emphasizes their role in enhancing model performance in identifying modified nucleotides such as 6mA.

Each representation acts as an orthogonal vector in a higher-dimensional space, which is a mathematical space with more dimensions than the original input features. This approach maintains the distinct characteristics of each nucleotide and provides a more flexible representation than one-hot encoding. Two essential parameters must be defined in the embedding layer:

- output_dim: This indicates the size of the vector space.
- input_length: This indicates the size of the input, corresponding to the window size.

### K-mer technique

Any DNA sequence can be split into consecutive fragments of length *k*, known as k-mers. K-mers are essential medical biomarkers for pathogen detection [35–37] and have been used in many studies related to DNA [38–40]. The choice of *k* can vary extensively based on the particular dataset and application. The k-mer model representing genetic sequences is defined as follows: let *S* be a genetic sequence of length *N*, represented as $L_{1},L_{2},L_{3},\ldots ,L_{M}$, where $L_{i}\in \{A,C,G,T\}$ for i=1,2,…,M. A string of *k* consecutive nucleotides in a DNA sequence is referred to as a k-mer. To determine the k-mers in a sequence, a sliding window of size *k* is applied, which moves one nucleotide at a time from position 1 to N−k+1, allowing for the entire sequence to be analyzed [41]. The choice to utilize a 1 nt step size stems from the necessity for high-resolution data when generating k-mers. This approach allows for the assessment of every possible k-mer combination within a sequence, thereby capturing subtle variations and patterns that are crucial in genomic analyses, where minor mutations can have significant biological implications.

While we acknowledge that advancing one nucleotide at a time demands greater computational resources, we explored larger step sizes (2 nt, 3 nt, and 4 nt) as alternatives. The comparative performance metrics for these approaches are summarized in Table 1.

**Table 1:** Performance metrics of k-mer analysis by step size.

Step Size (nt)	(ACC)	(SN)	(Sp)	(MCC)
1 nt	96%	96%	95%	0.92
2 nt	93%	90%	91%	0.85
3 nt	90%	86%	88%	0.75
4 nt	87%	83%	85%	0.65

The results reveal that the model employing a 1 nt step achieved an accuracy of 96%, surpassing the larger step sizes, which yielded accuracies of 93% for a 2 nt step, 90% for a 3 nt step, and 87% for a 4 nt step. Additionally, sensitivity declined with increasing step size, reflecting a notable loss of detection capability (1 nt: 96%, 2 nt: 90%, 3 nt: 86%, and 4 nt: 83%). Both specificity and Matthews Correlation Coefficient (MCC) underscore the advantages of the 1 nt approach, marking it as the superior option.

Despite the higher computational requirements of the 1 nt step, we believe that the enhancements in analytical precision and predictive power are justified, particularly in applications where accuracy is critical, such as disease detection and genetic analysis. Our decision to adopt a sliding window with a 1 nt step size was made after careful consideration of the trade-offs between computational demands and the need for a detailed high-resolution analysis of genomic sequences. The results strongly indicate that this method offers superior performance metrics, making it the preferred approach in our study.

To evaluate the different sizes of k-mer (k = 3, 4, 5, and 6), we rely on key performance metrics: Accuracy (ACC), Sensitivity (SN), Specificity (Sp), and Matthews Correlation Coefficient (MCC). Our assessment revealed that k = 3 produced the most favorable outcomes in all of these metrics. Specifically, the accuracy for k = 3 was recorded at 96%, outperforming larger k-mer sizes, k = 4 reaching 95. 11%, k = 5 at 94.50%, and k = 6 at 94.20%. Given that accuracy serves as a primary indicator of overall model performance, the superior accuracy of k = 3 validated its effectiveness.

In terms of sensitivity, k = 3 also demonstrated robust performance with a score of 96%, indicating the model’s reliability in accurately identifying positive instances, essential for applications requiring precise and timely detection. Furthermore, the specificity of the model k = 3 was 95%, reflecting its reliability in correctly classifying negative instances without misclassification, thus enhancing the efficiency of the model in distinguishing between different classes.

The Matthews Correlation Coefficient (MCC) for k = 3 was calculated at an impressive 0.92, demonstrating a strong overall performance considering all aspects of true and false positives and negatives. This balanced measure illustrated the predictive power of k = 3, significantly exceeding the other k-mer sizes, with k = 4 at 0.90, k = 5 at 0.89, and k = 6 at 0.88.

In summary, our analysis confirms that k = 3 provides the best results based on a thorough evaluation of accuracy, sensitivity, specificity, and MCC. Given these findings, in this study, we selected k = 3 after determining that it yielded the best results after our evaluations. For example, analyzing the sequence “ATCTATTTTTTTT...N,” we calculated the frequency distribution of each set of three consecutive nucleotides, shifting the window until the entire sequence was examined, resulting in a total extraction of 39 k mers. The results of our evaluation are summarized in Table 2

**Table 2:** Summary of evaluation results for different k-mer sizes.

Kmer size (nt)	(ACC)	(SN)	(Sp)	(MCC)
Kmer 3	96%	96%	95%	0.92
Kmer 4	95.11%	95.57%	94%	0.90
Kmer 5	94.50%	94.62%	93.90%	0.89
Kmer 6	94.20%	94.35%	93.84%	0.88

### Model architecture

The one-hot encoding model began by taking DNA sequences as input. This encoding technique represented each nucleotide with a distinct binary vector. The model processed these sequences through a convolutional layer with 128 filters, operating at a kernel size of 5 to extract local features. Next, a max pooling layer was utilized to reduce the dimensionality of the feature maps, highlighting the major characteristics of the data. Once the pooled output was flattened into a single vector, it transferred onto a dense layer that included 256 units. This was followed by a dropout layer set at a rate of 0.7 to minimize overfitting. The output successfully captured the learned features, but it did not yet incorporate the final output layer. The k-mer encoding model starts by taking the DNA sequences as input, similar to the one-hot encoding model. Conversely, instead of indicating individual nucleotides with distinct binary vectors, this model utilizes k-mer count vectors, which capture overlapping subsequences of a specified length (k). This approach allows for a comprehensive description of the sequence context, showing patterns that may be overlooked when concentrating on individual nucleotides. The first layer in this architecture is a dense layer with 128 units, which handles the k-mer data to identify crucial patterns. This is followed by a second dense layer that includes 256 units to extend and enhance the knowledge extracted from the k-mer counts. To reduce overfitting, a dropout layer with a 30% rate is utilized. Like the one-hot encoding model, the output produced after this dropout layer is rich in learned features but ignores the final output layer. For the ensemble learning model, the outputs from both the one-hot encoding model and the k-mer encoding model are averaged after their respective dropout layers. This averaging approach integrates the predictions from both models, thus minimizing individual biases and enhancing overall predictability. After this combination, a final dense layer with a sigmoid activation function is engaged to produce the ultimate binary classification output. This ensemble method efficiently leverages the strengths of both encoding techniques, enhancing the model’s ability to perform well in the classification task, as demonstrated by the performance measurements developed in this study. The parameters used in the model are shown in S1 Table. The models utilize the Adam optimizer, which adapts the individual learning rates for each parameter, allowing for efficient training and quicker convergence compared to classical stochastic gradient descent. The performance is evaluated using binary cross-entropy, which is defined mathematically as [34]:

$$-\frac{1}{N}\sum_{i=1}^{N}\left[y_{i}\log(p_{i})+(1-y_{i})\log(1-p_{i})\right]$$ (1)

where (y) is the true label and (p) is the predicted probability. The final node employs a sigmoid activation function, transforming the output into probabilities to determine the class label through a threshold (typically 0.5).

### Assessment of the model and performance indicators

In this study, we employed 10-fold cross-validation to evaluate the model’s effectiveness. This approach involves dividing the data into 10 equal segments. One segment is set aside for validation, while the model is trained on the remaining nine segments. This process continues until all segments have been used for validation. To ensure a consistent assessment of our model and to improve our understanding of the results, we assessed the following evaluation metrics: accuracy (ACC), sensitivity (SN), specificity (SP), and the Matthew’s correlation coefficient (MCC). These metrics were computed using the following equations:

$$\text{ACC}=\frac{TP+TN}{TP+TN+FP+FN}$$ (2)

$$\text{SN}=\frac{TP}{TP+FN}$$ (3)

$$\text{SP}=\frac{TN}{TN+FP}$$ (4)

$$\text{MCC}=\frac{TP\cdot TN-FP\cdot FN}{\sqrt{\left(TP+FP)(TP+FN)(TN+FP)(TN+FN\right)}}$$ (5)

In these calculations, the term TP (true positive) indicates the count of 6mA peptides that were accurately identified. TN (true negative) represents to the count of non-6mA peptides that were accurately identified. FP (false positive) denotes the instances in which non-6mA peptides were incorrectly classified as 6mA peptides, while FN (false negative) indicates cases in which 6mA peptides were misclassified as non-6mA peptides. Additionally, to evaluate the model’s capability to differentiate between 6mA and non-6mA sites, we calculated the area under the curve (AUC), which measures a classifier’s efficiency in discriminating between different classes. A greater AUC rate signifies excellent performance in distinguishing positive from negative samples, an AUC of 1 signifies the best classification of all 6mA and non-6mA samples, and an AUC of 0 signifies poor performance.

### Efficiency metrics and system specifications

In our analysis, we observed that the Average Runtime per Fold is 2.2028 seconds, while the Average Peak Memory Usage per Fold is 0.6510 MB. These metrics indicate that our model operates efficiently, exhibiting both low runtime and memory consumption, which are critical for practical applications, especially when processing large genomic datasets.

It is important to note that running time and memory usage are often overlooked in comparative studies in the literature, which typically emphasize accuracy, sensitivity, and other performance metrics. This lack of focus on computational efficiency can hinder meaningful comparisons between methods, as both runtime and memory efficiency are essential to deploy models in real-world scenarios.

By providing these metrics, we aim to highlight the practical applicability of our tool, enabling researchers to evaluate the model’s performance not only in terms of predictive accuracy but also regarding the efficiency of the predictions. This is particularly relevant for large-scale genomic analyses, where computational costs can significantly influence the feasibility of certain methodologies. The results of the analysis, including the system specifications and performance metrics, are detailed in Table 3.

**Table 3:** Runtime and memory usage metrics.

Metric	Value
Average Runtime per Fold	2.2028 seconds
Average Peak Memory Usage per Fold	0.6510 MB
CPU	11th Gen Intel(R) Core(TM) i9-11980HK @ 2.60GHz
Operating System	Windows 10, 64-bit OS
Installed RAM	32.0 GB
GPU	Nvidia GeForce RTX

These findings demonstrate the efficiency of our model and its potential applicability across various genomic analyses, paving the way for its broader use in the field.

## Results and discussion

### Implementation of feature type

The first dataset, referred to as Dataset 1, comprises sequences obtained from the Gene Expression Omnibus (GEO) of the National Center for Biotechnology Information (NCBI). This dataset contains sequences recognized for their N6-methyladenine (6mA) modifications, with an alteration score exceeding 30. To ensure quality, we used the CD-HIT clustering tool with a threshold of 60%, resulting in 880 positive instances. We also collected an equal number of negative instances based on strict criteria, including uniform sequence length and the presence of an unmethylated adenine in the center.

In contrast, Dataset 2 consists of approximately 265,290 sequences, providing a broader representation of k-mers. This dataset was analyzed with CD-HIT at an elevated threshold of 80% to eliminate duplicate sequences, yielding 154,000 verified 6mA sites. The increase in sample size and the diversity of sequences can lead to variations in how the model performs.

**Fig 3:**
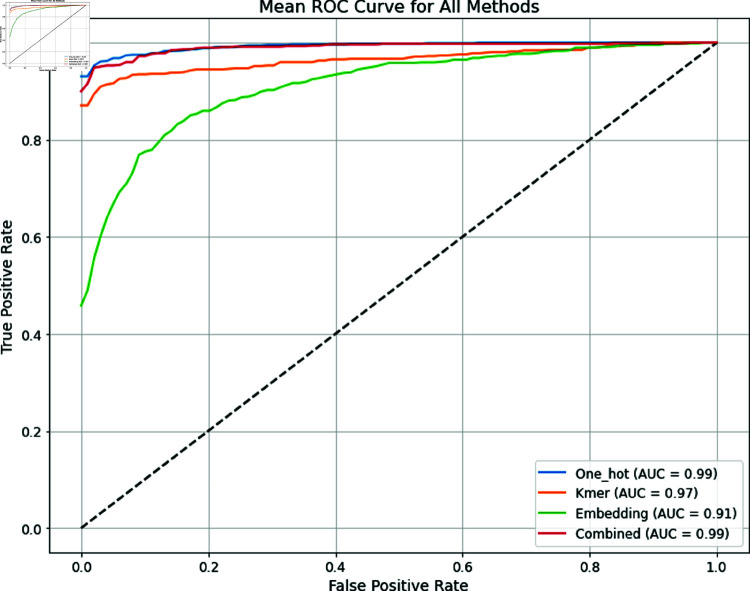
ROC curve comparison of individual methods versus combined method (one hot + kmer) using 10-fold cross-validation.

The assessment of numerous feature encoding techniques reveals important differences in their classification performance measurement, particularly in terms of accuracy (ACC), sensitivity (SN), specificity (SP), and the Matthew’s correlation coefficient (MCC). To detect the most effective grouping of feature types, we assessed the performance of several encoding methods, including one-hot encoding, embedding encoding, and k-mer encoding, alongside the top-performing grouping of features (one-hot encoding + k-mer encoding). The results of the 10-fold cross-validation assessment for each encoding method are summarized in Table 4. The performance comparison of all the methods based on the AUC is shown in Fig 3. One-hot encoding established strong performance among all metrics, reaching an accuracy of 97%, with both sensitivity and specificity measurements at 97%. A significant MCC value of 0.95 implies a robust relationship between the predicted and actual categories. These results indicate that one-hot encoding effectively captures the distinctions in categorical data, making it a reliable choice for tasks that require precise feature representation. This technique’s ability to denote each group as a binary vector confirms that it holds significant differences, which probably adds to its greater performance. In contrast, embedding encoding produced the lowest performance for the different measurements, with an accuracy of 82%, both sensitivity and specificity at 83%, and a remarkably lower MCC of 0.65. These outcomes raise questions regarding the efficiency of the embedding technique. The lower scores could indicate that this method either failed to sufficiently characterize the feature space or was not well suited for the specific descriptions of the dataset employed. This highlights the limitations of embedding methodologies when dealing with critical data, as they may not be as effective as more straightforward feature encoding methods. K-mer encoding performed estimably, achieving an accuracy of 96% and sensitivity and specificity of 96% and 95%, respectively, along with a powerful MCC of 0.92. These results are consistent with an existing study that showed that k-mer encoding is particularly effective in fields such as genomics and natural language processing, where sequence data are important [38, 39]. The k-mer method’s capacity to capture local patterns of sequences likely contributed to its strong performance, highlighting its utility in appropriate fields. The combined method of one-hot encoding and k-mer encoding produced the greatest performance in all measurements, with an accuracy of 98% and both sensitivity and specificity of 98%. The MCC value of 0.96 achieved proves the strength of this combined method. These superior results indicate that incorporating these encoding methods permits a more complete interpretation of the feature space, essentially leveraging the powers of each method. This interactive impact may enhance model execution by depicting categorical characteristics via one-hot encoding and serial dependencies among k-mer encoding.

**Table 4:** 10-fold cross-validation results comparing individual features with the optimal combined feature set using Dataset 1.

Feature Type	ACC	SN	SP	MCC
One-hot encoding	97	97	97	0.95
Embedding encoding	82	83	83	0.65
K-mer encoding	96	96	95	0.92
**Combined (one-hot encoding + k-mer encoding)**	**98**	**98**	**98**	**0.96**

In conclusion, the comparative evaluation of the feature encoding methods showed distinct variations in the performance results. While one-hot and k-mer encodings consistently delivered strong results, the performance of the embedding encoding method was comparatively lower. The benefits of the joint method highlight the capability of hybrid techniques to enhance feature demonstration and increase classification effectiveness, thus suggesting avenues for further research.

### Analysis of influential nucleotide motifs

We recognize the need to clarify the nucleotide sequences or motifs that significantly influence our classification study. To address this, we have utilized feature importance ranking to quantify the contributions of various input features.

In our revised analysis, we used a range of feature selection techniques, with their performance summarized in Table 5. Techniques like Recursive Feature Elimination (RFE) and Chi-square showed reasonable performance but were limited in capturing the full dataset’s potential. In contrast, the Convolutional Neural Network (CNN) approach using complete feature sets, especially those from one-hot and k-mer techniques, demonstrated enhanced accuracy.

**Table 5:** Performance comparison of feature selection techniques.

Feature Selection	ACC	SN	SP	MCC
PCA	80.28	79.71	80.84	0.61
Chi-square	84.26	82.73	85.62	0.68
RFE	84.49	83.84	85.06	0.69
Genetic Algorithm	78.52	77.40	79.51	0.57
All features	98	98	98	0.96

The CNN models’ ability to understand complex data patterns was highlighted as they accessed a more comprehensive feature landscape, allowing automatic capture of feature interactions and patterns, which may be overlooked by conventional selection methods. This robustness was evident as CNNs managed to filter out noise, isolating substantial biological signals.

To elucidate further, we examined the feature importance extracted from CNN models’ hidden layers, which pinpointed the ten most significant k-mer-based features, illustrated in S4 Fig. The visual representation in S4 Fig not only emphasizes influential motifs but also reinforces their biological implications in our classification study. Understanding the contributions of these motifs is vital for advancing our understanding of genomic sequences and their regulatory roles.

our comprehensive evaluation highlights the insights gained from leveraging feature importance analysis. It underscores traditional feature selection methods’ limitations while showcasing deep learning’s strengths in uncovering significant biological markers. Future research will continue to explore the implications of these motifs, in order to contribute further to genomic data analysis.

### Comparative performance analysis

The results from our model indicate differences in performance between the two datasets. In Dataset 1, we achieved an accuracy of 98.00%, while Dataset 2 yielded an accuracy of 95.00%. Several factors may influence these observations:

The larger dataset (Dataset 2) encompasses a wide range of sequences, introducing greater variability. Although this diversity can enhance the robustness of the model, it can also complicate feature identification, leading to a decrease in performance.

The criteria used to define positive and negative samples can impact the predictions of the model. In Dataset 1, strict criteria assisted the identification of specific motifs like AAAA and GAGG more effectively. In contrast, the larger and more varied structure of Dataset 2 could decrease the impact of individual motifs.

The different preprocessing methods, including selection thresholds, can affect the representation of characteristics. The varying similarity thresholds in the datasets might have resulted in the exclusion of key motifs from Dataset 2, impacting the classification accuracy.

### Comparison with other methods

In this study, we first performed a comparative assessment of the numerous predictor approaches by using Dataset 1 and a 10-fold cross-validation strategy, as displayed in Table 6. This technique not only permits a robust assessment of each method but also improves the consistency of the results achieved, acknowledging a significant comparison among distinctive methods.

**Table 6:** Comparison with different existing methods for Dataset 1 using 10-fold cross-validation.

Tools	SN	SP	ACC	MCC	AUC
iDNA6mA	86.7%	86.59%	86.64%	0.73	0.93
i6mA-DNCP	84.09%	88.07%	86.08%	0.72	0.93
MM-6mAPred	89.32%	90.11%	89.72%	0.79	-
6mA-Finder	-	-	-	-	0.94
SDM6A	85.2%	90.9%	88.1%	0.76	0.94
6mA-RicePred	84.89%	89.66%	87.27%	0.75	-
DNA6mA-MINT	94.25%	90.8%	92.53%	0.85	0.95
SpineNet-6mA	93.75%	95.79%	94.77%	0.89	0.98
i6mA-CNN	90.35%	94.62%	92.48%	0.85	0.98
Our method (DeepRice6mA)	**98%**	**98%**	**98%**	**0.96**	**0.99**

**Fig 4:**
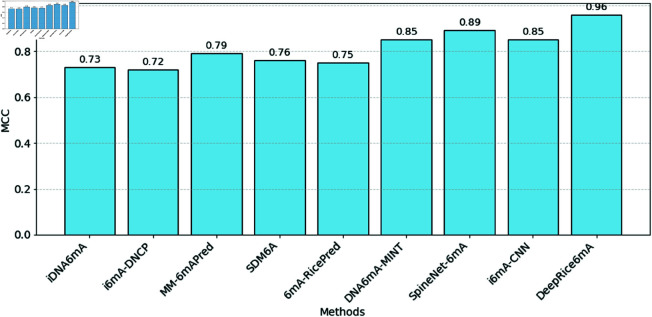
A bar plot illustrating the MCC values obtained through 10-fold cross-validation, comparing DeepRice6mA with existing methods.

**Fig 5:**
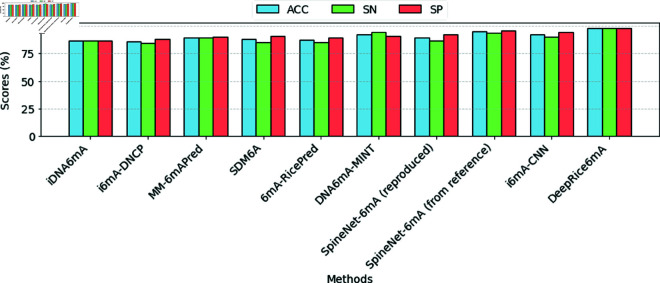
A bar plot illustrating ACC, SN, and SP values obtained through 10-fold cross-validation, comparing DeepRice6mA with existing methods.

With the evaluated tools, the merged method that employed both one-hot encoding and k-mer encoding appeared to be the most efficient. It reached outstanding performance metrics, containing sensitivity, specificity, and accuracy of 98% for all. This notable reliability throughout all fundamental implementation indicators positions the merged method as the foremost method in perfectly predicting the required results. Likewise, it obtained a Matthew’s correlation coefficient of 0.96 versus the existing methods, as depicted in Fig 4. The area under the curve was 0.99, demonstrating not only the technique’s ability but also its tremendous capacity to differentiate between classes. Fig 5 shows the performance of ACC, SN, and SP, respectively, against all the methods.

On the other hand, the i6mA-DNCP method showed the lowest performance measurements, with a sensitivity of 84.09%, a specificity of 88.07%, and an accuracy of 86.08%. Its corresponding Matthew’s correlation coefficient of 0.72 emphasizes noteworthy room for enhancement when compared to the top approaches. This drop in performance measurements highlights the need for an increase of higher-level methods or alterations to existing approaches to improve predictive abilities. Other methods, such as MM-6mAPred and 6mA-RicePred, also exhibited variability in their implementation. Notably, these methods did not define AUC values, which could complicate direct comparisons across all the assessed measurements. These gaps in the data indicate the essential boundaries in performance evaluations that demand additional exploration.

This study shows the distinct benefits of implementing the combined technique, especially due to its strong capability to steadily provide high quality predictions.

The outcomes underscore the importance of employing diverse encoding strategies to enhance model efficiency, thus reaffirming the value of this approach for future research. These findings set the stage for advancements in predictive modeling and significantly contribute to the existing knowledge in this field, establishing a comprehensive benchmark for subsequent investigations.

Additionally, the performance of various approaches was evaluated using the MCC scores obtained through the 10-fold cross-validation technique. The statistical evaluation demonstrated a significant difference among the methods, supported by an F-statistic of 726.32 and a p-value of $4.88\;\times \;10^{-72}$. As the p-value is considerably below the 0.05 threshold, we again confidently reject the null hypothesis, indicating that the methods do not produce equivalent results.

Furthermore, the performance of all methods was assessed using their Matthew’s correlation coefficient (MCC) scores, calculated through a 5-fold cross-validation approach. The statistical analysis revealed a significant difference in performance among the methods, evidenced by an F-statistic of 85.34 and a p-value of $2.82\;\times \;10^{-12}$. Given that the p-value is substantially lower than the threshold of 0.05, we can confidently reject the null hypothesis, indicating that the methods do not yield equivalent outcomes.

The substantial difference in MCC scores suggested by the high F-statistic implies that some methods outperform others in accurately predicting outcomes. Therefore, these results highlight that researchers should consider DeepRice6mA as the optimal choice for achieving superior predictive accuracy.

Additionally, we showed a comparative examination between our model and several existing methodologies using Dataset 2. All the methods employed the same dataset, and we used a consistent 5-fold cross-validation approach to confirm the validity of the outcomes. The performance metrics assessed included sensitivity (SN), specificity (SP), accuracy (ACC), Matthew’s correlation coefficient (MCC), and the area under the curve (AUC). The results of our evaluations are summarized in Table 7.

**Table 7:** Comparison with different existing methods for Dataset 2 using 5-fold cross-validation.

Tools	SN	SP	ACC	MCC	AUC
iDNA6mA-Rice	93%	90.5%	91.7%	0.83	0.96
SNNRice6mA	94.33%	89.75%	92.04%	0.84	0.97
SpineNet-6mA	95.71%	92.92%	94.31%	0.88	0.98
i6mA-CNN	95.13%	92.81%	93.97%	0.88	0.98
Our method (DeepRice6mA)	95%	95%	95%	0.90	0.99

**Fig 6:**
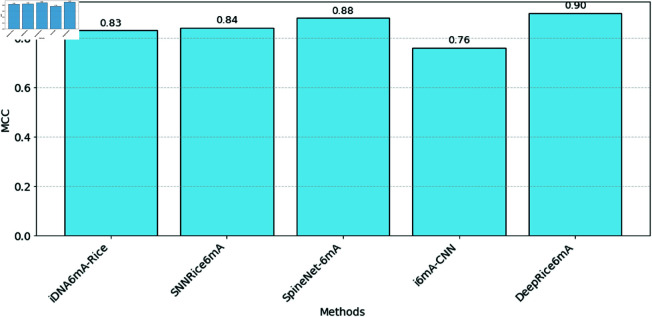
A bar plot illustrating the MCC values obtained through 5-fold cross-validation, comparing DeepRice6mA with existing methods.

**Fig 7:**
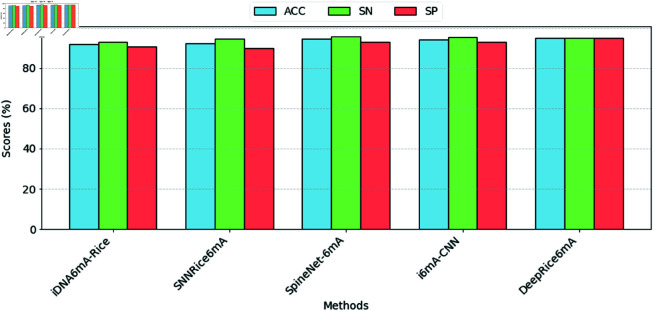
A bar plot illustrating ACC, SN, and SP values obtained through 5-fold cross-validation, comparing DeepRice6mA with existing methods.

The investigation emphasizes that our merged technique, integrating one-hot encoding and k-mer encoding, reached a significant level through all evaluated metrics. Specifically, it achieved a sensitivity of 95%, a specificity of 95%, an accuracy of 95%, and a Matthew’s correlation coefficient of 0.90 compared to recent methods, as displayed in Fig 6, and the area under the curve was 0.99. Fig 7 shows the performance of ACC, SN, and SP, respectively, against all the methods.

On the other hand, the lowest result among the existing methods was noted by iDNA6mA-Rice, which obtained a sensitivity of 93% and an accuracy of 91.7%. Although SNNRice6mA showed slightly better measurements, with a sensitivity of 94.33% and an accuracy of 92.04%, both approaches fell short of our combined technique’s performance. Furthermore, although SpineNet-6mA and i6mA-CNN showed reasonable results with equally high sensitivity and specificity scores, they did not score at the same level of overall accuracy as our proposed model. Overall, these outcomes demonstrate the efficiency of our approach, determining it to be the best current method compared to the earlier tested methods.

### Evaluating performance on diverse test sets

The model was trained specifically on rice genome data, which limits its direct applicability to other plant species. However, the methodologies underlying DeepRice6mA are inherently adaptable for application to other genomes, such as maize and wheat. To demonstrate this adaptability, we suggest using transfer learning techniques, in which the model initially trained on rice can be fine-tuned with additional datasets from maize and wheat. This approach leverages the learned parameters and can significantly enhance the model’s performance on these new genomes.

An independent test set is essential to verify the generalizability of a method. In our study, we applied three distinct test datasets to assess the reliability of our proposed tool to identify N6-methylated sites within plant genomes. We obtained sequences containing 6mA sites from Fragaria vesca and Rosa chinensis from the MDR database [42], while sequences for Arabidopsis thaliana were retrieved from the NCBI GEO under accession number GSE81597 [43]. Only sequences with an adenine nucleotide in the middle and an alteration score of 30 or higher were retained. CD-HIT software was then employed to remove sequences with 80% or greater similarity.

The results presented in Table 8 demonstrate the ability of our tool to accurately identify 6mA sites in different plant species. Specifically, the tool achieved impressive accuracy rates of 97. 97%, 96. 95%, and 87. 83% when evaluated in data sets from Fragaria vesca, Arabidopsis thaliana, and Rosa chinensis, respectively. In particular, our tool surpasses the performance of the i6mA-CNN tool across all species in terms of success rates, highlighting its superior predictive capability in the context of identifying nucleotide modifications.

**Table 8:** Performance of DeepRice6mA in identifying 6mA sites across different plant genomes.

Plant genome	Number of samples	Number of correct predictions	Success rate (%)
Fragaria vesca	8,979	8,780	97.97
Arabidopsis thaliana	67,650	64,800	96.95
Rosa chinensis	1,126	966	87.83

For future validation, we recommend implementing rigorous independent testing protocols using publicly available datasets for these species. This could include comparisons of the predictions made by DeepRice6mA with those from established methods. By validating our tool against diverse genomes, we can ascertain its robustness and generalizability.

In conclusion, these steps will not only establish the efficacy of DeepRice6mA across multiple species but also provide valuable insights into the understanding of 6mA site identification in diverse plant genomes, paving the way for more comprehensive applications in plant genomics.

### Detected likely motifs

In deep learning, motifs are distinct patterns that neural network algorithms aggressively request to improve the accuracy of identification tasks. In our investigation of the identification of the 6-methyladenine (6mA) site using benchmark datasets, we discovered a range of probable motifs, which are specified in Tables 9 and 10. Furthermore, experimental validation confirmed previous findings that the 6mA modification is primarily located within GAGG motifs. Our analytical method also revealed that GAGG is the third prominent motif utilized by our model, that is a deep learning-based model designed to identify 6mA sites in genomic sequences. A comparison with previous research conducted by Rahman *et al*. [20], reveals consistency in motif identification between our findings and their study. In Rahman’s exploration, operating dataset 1 with a 10-fold cross-validation approach, the most predominant motifs included AAAA (53%), TTTT (34%), AAGA (29%), and GAGG (29%). Furthermore, in a subsequent analysis using Dataset 2 with 5-fold cross-validation, a similar trend emerged, with AAAA (55%), GAGG (37%), TTTT (34%) and AAAT (32%) being the most frequent motifs. These results suggest a significant correlation between our findings and Rahman’s findings on the dominance of AAAA and GAGG motifs in both datasets. This alignment implies that these motifs are fundamental in the context of the 6mA site identification and are therefore worthy of future investigation.

**Table 9:** Motif frequency analysis using Dataset 1 with 10-fold cross-validation.

Motif	Occurrence (%)
AAAA	53%
TTTT	34%
AAGA	29%
GAGG	29%

**Table 10:** Motif frequency analysis using Dataset 2 with 5-fold cross-validation.

Motif	Occurrence (%)
AAAA	55%
GAGG	37%
TTTT	34%
AAAT	32%

The AAAA motif is frequently located in promoter regions and regulatory elements, where it is vital for controlling gene expression. Adenine-rich sequences affect chromatin accessibility by influencing nucleosome positioning, subsequently facilitating the binding of transcription factors. [44]. In Arabidopsis thaliana , adenine homopolymers are particularly abundant in regions that are epigenetically regulated, including sites modified by N6-methyladenine (6mA). [43]. The observation of AAAA motifs in these regions implies that they could serve as recognition sites for 6mA methyltransferases, thus contributing to epigenetic regulation of gene expression.

The GAGG motif has been recognized as a potential epigenetic marker, especially in genomic regions enriched with N6-methyladenine (6mA) modifications. Research in Arabidopsis thaliana suggests that the GAGG motif is prevalent and may indicate a conserved mechanism that involves the roles of 6mA writers, readers, and erasers throughout evolutionary processes [45].

Although the TTTT motif lacks adenine, it has a substantial impact on the dynamics of the DNA structure, which may indirectly influence 6mA deposition and function. Poly-thymine sequences contribute to the formation of single-stranded loops and secondary DNA structures, affecting transcription termination, replication efficiency, and chromatin accessibility [44]. Within plant genomes, TTTT motifs are often found in noncoding regulatory regions, where they might interact with DNA-binding proteins to modulate gene expression. Although direct evidence linking TTTT motifs to 6mA modifications remains limited, their abundance in regulatory elements suggests a role in epigenetic regulation by altering the structure of chromatin and increasing DNA accessibility [45].

The identified motifs are functionally significant in regard to 6mA modifications, enriched in regulatory regions, transposable elements, and chromatin accessible sites, where they can influence the recognition, deposition, and function of 6mA. The AAAA motif is correlated with chromatin accessibility and transcriptional activation, while the GAGG motif is related to proteins involved in gene regulation and methylation. The TTTT motif may contribute to structural changes that affect chromatin accessibility. These insights improve our understanding of the biological significance of these motifs within plant genomes.

Additionally, while it might initially appear that the k-mer approach, which focuses on 3-nucleotide (nt) sequences, conflicts with the identification of 4-nt motifs such as “TTTT” and “AAAA,” it is crucial to recognize that the k-mer analysis provides a foundational framework. This framework allows for the extraction of insights from shorter sequences and facilitates the identification and exploration of longer motifs that have biological relevance.

Regarding the relationship between the motifs “TTTT” and “AAAA” in relation to identical genomic regions or reverse complement sequences, our analyzes reveal that while they may exhibit complementary characteristics in specific contexts, they reflect distinct functional roles within the genomic landscape. This differentiation highlights the complexities involved in motif significance and their potential interactions.

## Conclusion

In this study, we developed DeepRice6mA, a novel computational tool proposed to identify N6-methyladenine (6mA) sites in the rice genome. By utilizing a deep learning method that combined one-hot encoding with 3-kmer feature encoding, DeepRice6mA exceeded existing techniques in both accuracy and computational effectiveness. Our method reached state-of-the-art performance, with a 98% accuracy percentage, along with sensitivity, specificity, Matthew’s correlation coefficient (MCC), and area under the receiver operating characteristic curve (AUC) grades of 98%, 98%, 0.96, and 0.99, respectively, based on ten-fold cross-validation.

While earlier approaches have revealed promise, they face restrictions, such as element bias and unsatisfactory predictive implementation for large-scale genomic tools. Our work highlights the importance of employing consistent computational methods when studying epigenetic alterations, particularly in addressing the challenges associated with experimental detection techniques. We believe that the motifs recognized through DeepRice6mA can deliver valuable understandings for further study into 6mA and its genetic consequences.

Looking ahead, our goal is to enhance DeepRice6mA by increasing our dataset and improving the model with extra feature sets and superior deep learning architectures. We are confident that DeepRice6mA provides a valuable resource for the scientific community, improving our ability to understand and analyze DNA methylation changes. The source code and dataset will be publicly available to assist continued research in this fundamental area of analysis.

## Supporting information

S1 Table Hyperparameters of One-hot encoding model. (PDF)

S2 Table Hyperparameters of K-mer encoding model. (PDF)

S3 Table The average accuracy (Acc), MCC, sensitivity (Sn), and specificity (Sp) of 10-fold Cross Validation results for different window sizes. (PDF)

S4 Fig The most influential features identified from the hidden layers of the CNN model. (TIFF)
