# Supplementary material for: DeepRice6mA: A convolutional neural network approach for 6mA site prediction in the rice Genome
Source: PLoS One. 2025 Jun 18;20(6):e0325216. doi: 10.1371/journal.pone.0325216 (PMC12176223; doi:10.1371/journal.pone.0325216)
Supplement: S1 Table — (PDF) [file pone.0325216.s001.pdf]

S1 Table: Hyperparameters of One-hot encoding model

**Parameters**	**Settings**
**Learning Rate**	0.001
**Batch Size**	64
**Epochs**	50
**Convolutional Layer Type**	Conv1D
**Convolutional Filters**	128
**Kernel Size**	5
**Pooling Layer Type**	MaxPooling1D
**Pooling Size**	2
**Dropout Rate**	0.7
**Dense Layer Units**	256
