# Supplementary material for: DeepRice6mA: A convolutional neural network approach for 6mA site prediction in the rice Genome
Source: PLoS One. 2025 Jun 18;20(6):e0325216. doi: 10.1371/journal.pone.0325216 (PMC12176223; doi:10.1371/journal.pone.0325216)
Supplement: S2 Table — (PDF) [file pone.0325216.s002.pdf]

S2 Table: Hyperparameters of K-mer encoding model

**Parameters**	**Settings**
**Learning Rate**	0.001
**Batch Size**	64
**Epochs**	50
**Dense Layer 1 Units**	128
**Dense Layer 2 Units**	256
**Dropout Rate**	0.3
