# Supplementary material for: DeepRice6mA: A convolutional neural network approach for 6mA site prediction in the rice Genome
Source: PLoS One. 2025 Jun 18;20(6):e0325216. doi: 10.1371/journal.pone.0325216 (PMC12176223; doi:10.1371/journal.pone.0325216)
Supplement: S3 Table — (PDF) [file pone.0325216.s003.pdf]

S3 Table: The average Accuracy (Acc), MCC, Sensitivity (Sn), and Specificity (Sp) of 10-fold Cross Validation results for different window sizes.

Window sizes	Acc	MCC	Sn	Sp
31	0.76	0.45	0.77	0.74
33	0.80	0.52	0.79	0.75
35	0.83	0.58	0.82	0.76
37	0.86	0.66	0.85	0.79
39	0.91	0.84	0.92	0.87
**41**	**0.98**	**0.96**	**0.98**	**0.98**
43	0.94	0.90	0.93	0.91
45	0.89	0.83	0.88	0.86
47	0.84	0.78	0.84	0.81
49	0.82	0.72	0.81	0.76
