# Supplementary figures and images for: DeepRice6mA: A convolutional neural network approach for 6mA site prediction in the rice Genome
Source: PLoS One. 2025 Jun 18;20(6):e0325216. doi: 10.1371/journal.pone.0325216 (PMC12176223; doi:10.1371/journal.pone.0325216)

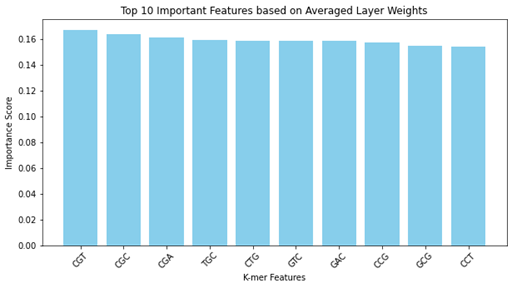

Supplement: S4 Fig — (TIFF) [file pone.0325216.s004.tif]
